# Low Molecular Weight Chitosan–Insulin Polyelectrolyte Complex: Characterization and Stability Studies

**DOI:** 10.3390/md13041765

**Published:** 2015-03-30

**Authors:** Zakieh I. Al-Kurdi, Babur Z. Chowdhry, Stephen A. Leharne, Mahmoud M. H. Al Omari, Adnan A. Badwan

**Affiliations:** 1 The Jordanian Pharmaceutical Manufacturing Company (PLC), Suwagh Subsidiary for Drug Delivery Systems, P.O. Box 94, Naor 11710, Jordan; E-Mails: zkurdi@jpm.com.jo (Z.I.A.-K.); momari@jpm.com.jo (M.M.H.A.O.); 2 Faculty of Engineering & Science, University of Greenwich, Medway Campus, Chatham Maritime, Kent ME44TB, UK; E-Mails: b.z.chowdhry@greenwich.ac.uk (B.Z.C.); s.a.leharne@greenwich.ac.uk (S.A.L.)

**Keywords:** insulin, oligochitosan, low molecular weight chitosan, polyelectrolyte complex, stability

## Abstract

The aim of the work reported herein was to investigate the effect of various low molecular weight chitosans (LMWCs) on the stability of insulin using USP HPLC methods. Insulin was found to be stable in a polyelectrolyte complex (PEC) consisting of insulin and LMWC in the presence of a Tris-buffer at pH 6.5. In the presence of LMWC, the stability of insulin increased with decreasing molecular weight of LMWC; 13 kDa LMWC was the most efficient molecular weight for enhancing the physical and chemical stability of insulin. Solubilization of insulin-LMWC polyelectrolyte complex (I-LMWC PEC) in a reverse micelle (RM) system, administered to diabetic rats, results in an oral delivery system for insulin with acceptable bioactivity.

## 1. Introduction

One of the most challenging problems in the development of liquid peptide/protein pharmaceuticals is their physical and chemical instability. Most peptides and proteins are formulated so that they can be administered clinically by parenteral injections, as this is the fastest route towards commercialization. However, stabilization of peptides and proteins in a designated delivery system against degradation, particularly in the gastrointestinal tract (GIT), is a prerequisite for oral delivery. This can be carried out by using several excipients such as salts, amino acids, surfactants, polyhydric alcohols, and carbohydrates [1]. The latter include chitosans, which are composed of β-(1-4)-linked d-glucosamine and *N*-acetyl-d-glucosamine units [2]. Chitosans are non-toxic, degradable, and biocompatible polymers that exhibit different characteristics. This allows them to be used as excipients for protein formulations intended for *in vivo* delivery of biopharmaceuticals [3].

The presence of free NH_2_ groups allows chitosan to form polyelectrolyte complexes (PECs) with negatively charged moieties. For example, PECs containing chitosan, alkyl-chitosan, and PEG-grafted alkyl-chitosan have been shown to improve insulin delivery [4,5]. Such PEC nanoparticles showed a pH-dependent stabilization of insulin [6].

Schatz *et al*. synthesized a partially *N*-sulfated chitosan. Upon acidification, nanoparticles were formed by electrostatic interactions between the non-sulfated protonated amine groups of chitosan and the negatively charged *N*-sulfated chitosan amines. These PECs can be used for encapsulation of macromolecules [7].

However, the main function of such PECs is to increase protein stability towards harsh conditions in the GIT or unfavorable storage conditions and to protect them against physical and chemical instabilities. PEC nanoparticles prepared by Mao *et al*. using chitosan, trimethyl-chitosan, PEGylated-trimethyl-chitosan, and insulin were unaffected by lyophilization and PECs were shown to protect insulin from degradation even at temperatures as high as 50 °C for 6 h [8]. LMWCs have been used to prepare the PECs employing poly-γ-glutamic acid, which was used as a carrier for insulin, followed by enteric coating or layering with calcium alginate in order to allow oral administration [9]. Jintapattanakit *et al*. found that PECs prepared using trimethyl-chitosan (100 kDa) and PEG-graft-trimethyl-chitosan copolymer improve the stability of oral insulin [10]. Also, Song *et al*. reported an oral insulin delivery system based upon ultrathin nanofilm encapsulation technology. The proposed system can be used to load a high amount of insulin (90%) using chitosan (l50–190 kDa, 75% deacetylation) [11]. Additionally, it has also been reported that the inclusion of chitosan in lipid nanoparticles enhances the physical stability of insulin by protecting against proteolysis [12].

A novel system based on solubilization of the insulin–chitosan PEC in RM system made from PEG-8 caprylic/capric glycerides and glycerol-6-dioleate as emulsifying agents and dispersed in oleic acid, has been patented by Badwan *et al.* [13,14]. The function of this solubilized PEC is to reduce the size of particles intended for oral delivery of insulin [15].

Generally, studies have been undertaken by using high molecular weight chitosans, and in the vast majority of cases the chemical stability of insulin was not evaluated in term of insulin degradation and formation of its degradation products. The objective of the work reported herein was to investigate the influence of complexing LMWCs of different molecular weights (1.3–30 kDa) with insulin on its physicochemical and biological stabilities. Insulin content was monitored and examined as well as the formation of high molecular weight proteins (HMWPs) of insulin. Furthermore, the system was evaluated for bioactivity.

## 2. Results and Discussion

### 2.1. HPLC Methods Verification

Insulin (I), A-21 desamido insulin, and HMWPs were determined by using the assay and limit of HMWP tests as stated in the human insulin USP monograph [16]. The insulin assay method was adopted for evaluating insulin stability in bulk insulin powder and in injectables [17]. Additionally, the method was found to be convenient for evaluating the stability of insulin in other delivery systems [18].

In the present work, the suitability of USP HPLC methods for the determination of insulin and HMWP in I-LMWC PEC systems was examined. Practically, insulin may be recovered either by an extraction-based method with organic solvents or by hydrolysis of the carriers (PEC or RM) with an alkaline reagent [19]. In the current work, different extraction solvents and procedures to extract insulin were evaluated. Aqueous solutions (0.01 or 0.1 N HCl) and methanol at different ratios (1/1, 2/1, 1/2, 2/3, and 3/2 v/v) were used to obtain the optimal ratio of the extraction solvent mixtures. Extraction of insulin from I-LMWC PEC preparations with a mixture of 0.01 M HCl and methanol at a ratio of 2/3 (v/v) gave acceptable recoveries for insulin (>98%); moreover, the stability of insulin in such a mixture was retained for 24 h.

Furthermore, the USP HPLC method parameters used for the assessment of the stability of bulk insulin, such as the ratio of mobile phase components and temperature [16], were evaluated for the PEC and RM systems. The ratio of mobile phase components (aqueous to acetonitrile) was found to be critical as a high acetonitrile content and a decrease in column temperature (e.g., to 35 °C) led to the precipitation of sulfate salt and subsequently affected the HPLC system parameters (e.g., retention time of insulin and resolution).

Verification of the USP HPLC method for insulin showed a linear response for signal output *versus* insulin concentration over the concentration range of 0.9–10 mg insulin/mL with an *R*^2^ value >0.995; such results are in line with the acceptable verification limits [20]. The intra- and inter-day relative standard deviation (RSD) values were less than 2%, indicating good precision. No interfering peaks from the components of the delivery systems were detected. The resolution factor between insulin and A-21 desamido insulin was >2.0, indicating that the method is specific. The method sensitivity was proved by low detection limit (DL) (0.02 mg/mL) and quantitation limit (QL) (0.08 mg/mL) values. Thus, the isocratic HPLC method developed herein is, analytically, advantageous in comparison with the published gradient methods [21,22]. The results of verification of the HPLC method confirmed the applicability of the USP HPLC method for the analysis of preparations other than injectables, such as polyelectrolyte systems. Consequently, the USP method can be considered as stability indicating method for the analysis of PEC and RM delivery systems.

**Figure 1:**
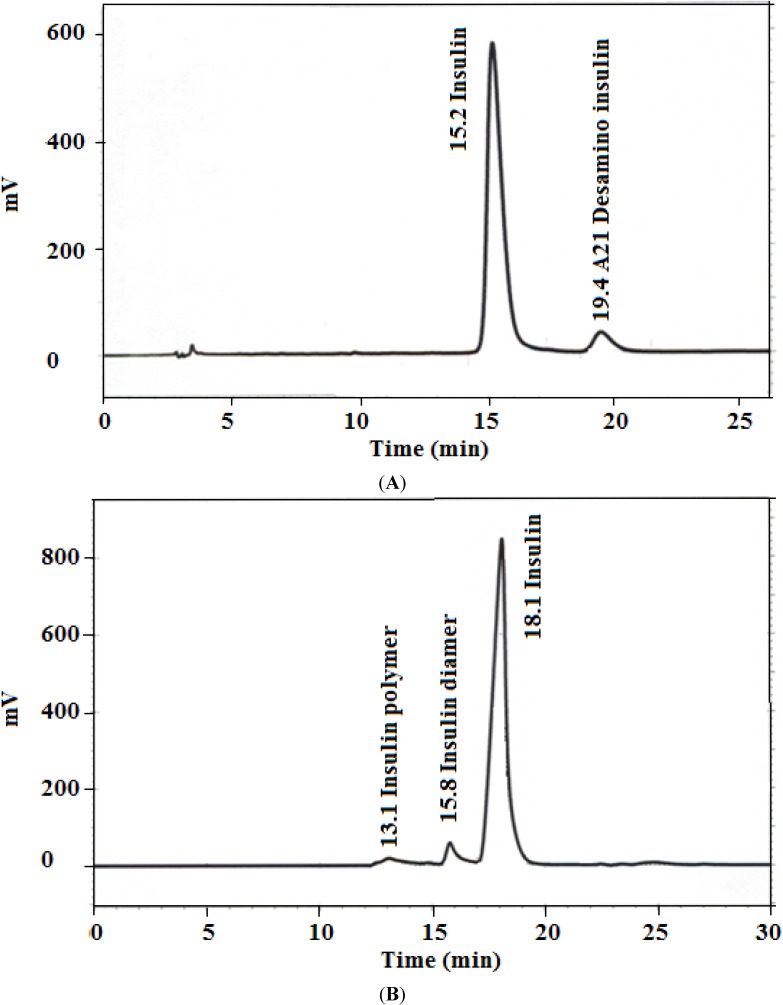
Representative HPLC chromatograms for (**A**) insulin assay (1.5 mg/mL) and (**B**) high molecular weight proteins (HMWPs) limit test (4.0 mg/mL).

The data in Figure 1 shows representative HPLC outputs for both assay and limit of HMWP tests, where the peaks corresponding to insulin, A-21 desamido insulin (Figure 1A), and HMWPs (Figure 1B) are well resolved. The results of HPLC method verification are summarized in Table 1; all the verification parameters are within acceptable limits [20].

**Table 1:** HPLC method verification results for insulin assay.

Analytical Parameter	Result
Linearity Range	
- 0.9–10.0 mg/mL	*R*^2^ = 0.9998
Specificity	
- Interference	No interference from formulation components and related compounds
Precision (%)	
- Inter-day RSD (*n* = 3 × 3)	0.8–1.1
- Intra-day RSD (*n* = 5)	0.7–0.9
Recovery (%)	98.3 ± 0.9
DL (mg/mL)	0.02
QL (mg/mL)	0.08
Stability of Solution	
- Decrease in assay at ambient condition	2.5% decrease in assay after 24 h
System Suitability	
- Resolution between insulin and A-21 desamido insulin	3.7
- Tailing factor for insulin peak	<1.8
- RSD of replicate injections	<1.6%

DL and QL: detection and quantitation limits, RSD: relative standard deviation.

### 2.2. Effect of the Molecular Weight and Concentration of LMWC on Insulin Stability

Prior to studying the effect of the molecular weight of LMWC on the stability of insulin, different parameters including insulin concentration (0.9–10.0 mg/mL) and solvent (water, phosphate buffer pH 6.5, and Tris-buffer of pH 6.5), were investigated at 50 °C for 72 h. It was found that the stability of insulin increases when the concentration of insulin is increased; these results agree well with reported data [23,24,25,26]. However, it appears from the results of the present study that a concentration of 7 mg/mL is sufficient for maintaining insulin stability at pH ≈ 6.0. Furthermore, Tris-buffer (an organic buffer) improved the stability of insulin even at low concentrations of insulin (0.9 and 1.75 mg/mL) by inhibiting the re-aggregation and precipitation of insulin. Also, at neutral pH, the conversion of monomeric insulin to form dimeric, tetrameric, and eventually hexameric insulin seems to stabilize insulin [27]. At insulin concentrations of 0.9–10 mg/mL, the fractional content of the degradation products A-21 desamido insulin and dimer did not exceed 0.4% after incubation at 50 °C for 72 h. On the other hand, other HMWPs were not detected over the investigated range of insulin concentration. In neutral solutions, the insulin molecules are associated mainly into non-covalent, Zn^2+^-containing hexamers [28]. The observation that dimer formation is independent of insulin concentration indicates that the intermolecular chemical reaction occurs mainly within the hexameric units and not between the hexamers in solution [29]. This might be due to the fact that hexamers are less susceptible to degradation [23].

**Figure 2:**
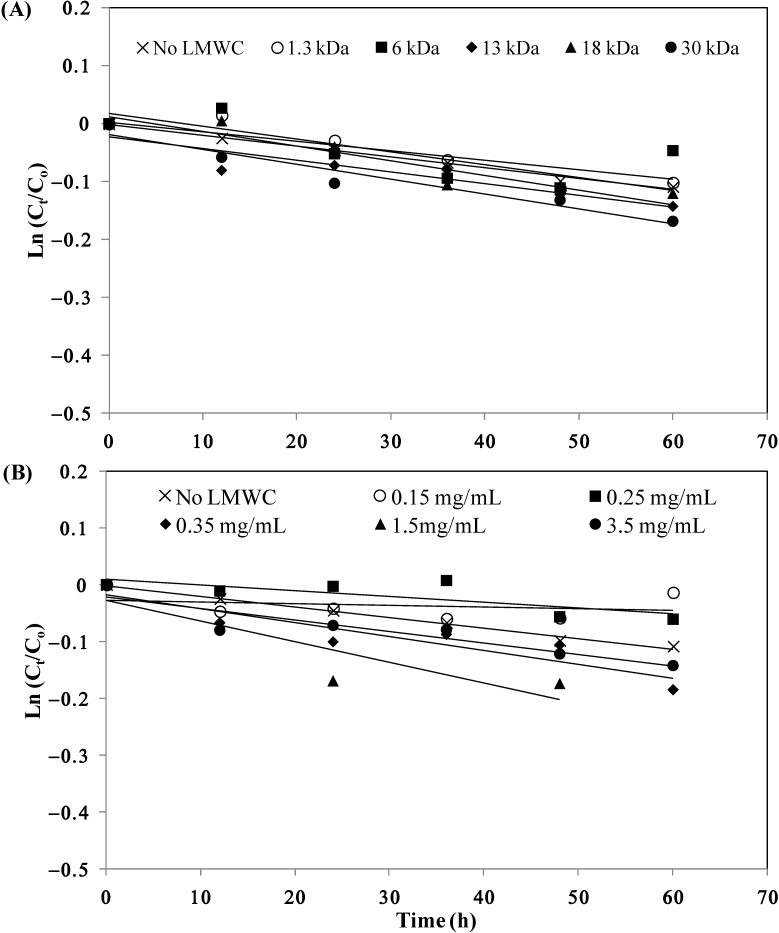
Stability of insulin in the presence of (**A**) different LMWCs (1.5 mg/mL) and (**B**) different concentrations of 13 kDa LMWC at 50 °C. C_o_ is the initial insulin concentration and C_t_ is the insulin concentration at different periods of incubation time. The values of Ln (C_t_/C_o_) are the mean of 3 replicates for each experiment (RSDs < 5%).

The stability of I-LMWC PECs prepared by using LMWCs of different molecular weights (1.3–30 kDa) at 50 °C is shown in Figure 2A. The kinetic parameters for insulin degradation, as shown in Table 2, indicate a noticeable effect of molecular weight of LMWC on the extent of insulin degradation in the I-LMWC PEC preparations (*i.e.*, stability decreases with increasing molecular weight of LMWC).

**Table 2:** First-order kinetic parameters for insulin–LMWC polyelectrolyte complex (I-LMWC PEC) degradation in the presence of different LMWCs and different concentrations of 13 kDa LMWC at 50 °C.

LMWC Molecular Weight (kDa) *^,+^	Insulin/LMWC Molar Ratio	*k* (h^−1^) × 10^3^	*t*_90_ (h)	*t*_50_ (h)	*R*^2^
0.0	-	1.2	88	587	0.2065
1.3	1/4.5	2.2	48	315	0.8780
6	1/1	1.6	64	423	0.4835
13	1/0.45	2.0	52	344	0.8428
18	1/0.3	2.5	42	274	0.8831
30	1/0.2	2.6	41	272	0.9388
**[13 kDa LMWC] (mg/mL) ^+^**					
0.0	-	1.2	88	587	0.2065
0.15	1/0.02	1.3	81	533	0.9200
0.25	1/0.03	1.0	102	675	0.5871
0.35	1/0.045	2.4	43	283	0.8388
1.5	1/0.2	3.6	29	191	0.7704
3.5	1/0.45	2.0	52	344	0.8428

*k*: 1st order rate constant, *t*_90_ and *t*_50_: shelf-life for 90% and 50% intact potencies. **^+^** [Insulin] is 3.5 mg/mL, * [LMWC] is 3.5 mg/mL.

The effect of 13 kDa LMWC concentration on the stability of I-LMWC PECs at 50 °C is shown in the data in Figure 2B. The results revealed a noticeable effect of increasing LMWC concentration on the degradation of insulin (Table 2).

In addition, monitoring the physical stability of I-LMWC PEC indicated that the formation of a physically stable system depends upon the molecular weight of LMWC. For example, LMWCs of shorter chains formed soluble complexes, while turbidity and precipitation were observed with higher molecular weight LMWCs (e.g., 30 kDa). This can be explained by the fact that complex formation between insulin and LMWC is mainly governed by kinetic factors which leads to preferential binding with the shorter chains due to their flexibility [8]. On the other hand, the chemical stability of insulin was affected to the same extent in the presence of different molecular weight LMWCs (Figure 2A). This may be attributed to the ratio of ionized groups of insulin (7.0 × 10^20^) to chitosan (5.3 × 10^21^), which was fixed at about 1/8 in the preparation of different I-LMWC PECs by changing the molar ratio of insulin: LMWC 1/0.2–1/4.5 (Table 2). The number of ionizable groups was calculated on the basis of previously published work [15].

Furthermore, changing the concentration of 13 kDa LMWC showed a significant effect on insulin stability (Figure 2B). Insulin instability was observed mainly at high concentrations (3.5 mg/mL), while at lower concentrations the effect is less. This may be explained by the changes in LMWC structure and particle size with concentration. With increasing concentration of LMWC, the structure becomes more helical, leading to the formation of aggregates and a concomitant increase in particle size (see Section 2.4). At high concentration, such aggregates may prevent LMWC from protecting insulin (Figure 2B).

By monitoring the degradation of insulin as a function of molecular weight of LMWC, the fraction of A-21 desamido insulin (0.2% initial) increases when the molecular weight of LMWC is increased after 72 h incubation at 50°C (0.9, 1.1, 1.2, 1.4, and 1.6% for 1.3, 6, 13, 18, and 30 kDa LMWC, respectively). While the results showed an increase in the content of insulin dimer (from 0.1% to 0.6%–0.7%) regardless of the molecular weight of LMWC. This may be attributed to the fixed ratio of ionized groups of insulin to LMWC. The amino groups of LMWC have the capacity to react with insulin by intermolecular aminolysis, resulting in transamidation between the molecules. Such a reaction can result in dimer formation [29].

However, the formation of other HMWPs increases as the molecular weight of the LMWCs increases (1% for 1.3 kDa and 3%–4% for 6–30 kDa LMWCs). The formation of dimers and polymers can be explained by the ability of free NH_2_ groups to react with the carbonyl groups of insulin (available in Asn at A-21 and B-2, and Gln available at A-5, A-15, and B-4) by intermolecular aminolysis, resulting in transamidation or Schiff base-mediated reactions between molecules forming HMWPs and, in parallel, dimer formation, probably as a result of disulfide interchange [29]. It is worth mentioning that the impact of HMWP formation on the quality and therapeutic usefulness of the pharmaceutical preparation is attributed to safety rather than efficacy. Preparation efficacy will not be affected by a decrease in the content of insulin. However, some of the immunological side effects associated with insulin therapy may be due to the presence of covalent aggregates of insulin in the therapeutic preparations, and specific antibodies against dimers have been identified in 30% of insulin-treated diabetic patients [30]. Dimer levels of 2% generated a highly hypersensitive response [31]. Accordingly, the content of HMWPs should be kept as low as possible. The acceptable USP limit does not allow more than 3.0% HMWPs in insulin pharmaceutical preparations. This indicates that during the development of insulin delivery systems, optimization of the system is essential to prevent the formation of HMWPs.

### 2.3. Effect of the Molecular Weight of LMWC on Immunological Bioactivity of Insulin

The ELISA method used for insulin determination was found to be linear in the range of 0–100 µIU/mL with an R^2^ value of 0.868 and DL of 0.26 µIU/mL. Method recovery at the recommended concentration (by the kit supplier), 50 µIU/mL, was carried out; the fraction recovered was 92.7%, with an RSD of 6.1% (average of 3 samples). Monitoring of insulin solutions incubated at 50 °C indicated a loss in bioactivity of 40% after an 8 h incubation, which indicates that the method is specific and loss in bioactivity of insulin can be detected using ELISA. The ELISA results indicated that insulin in the different I-LMWC-PECs prepared with LMWC of different molecular weights (1.3–30 kDa) retained its bioactivity (assay > 85%) (Table 3). The ELISA method has very high sensitivity and specificity as it depends on the reaction of the predominant protein with a specific antibody to form a complex [32]. Therefore, ELISA antigenicity is considered to be an appropriate means for detection of changes of insulin antigenic activity [33]. The surface structure of insulin was assessed by antibodies that bind to the epitopes on the insulin. The ELISA results indicated that insulin in the PEC had retained its bioactivity. ELISA results were comparable with HPLC results, which were used to assess the integrity of insulin and confirmed that the receptor-binding epitopes on insulin were maintained after complexation with LMWC [34].

**Table 3:** The immunological bioactivity of insulin-LMWC polyelectrolyte (I-LMWC PEC) of different molecular weight after incubation at 50 °C for 72 h.

LMWC Molecular Weight (kDa)	Bioactivity (%)	RSD
No LMWC	58.9 *	10.3
1.3	91.1	3.1
6	92.3	4.7
13	102.9	6.7
18	94.6	6.1
30	97.1	5.7

* After 8 h of incubation, RSD: relative standard deviation.

The aforementioned stability results show a profound impact of molecular weight (*i.e.*, chain length), concentration (*i.e.*, charge ratio), pH, and buffer type of the LMWC on the development of a stable oral delivery systems for insulin. In the present work, the 13 kDa LMWC can be considered a suitable candidate to prepare the PEC and the RM systems. It showed an optimal formulation with respect to chemical and physical stability. Furthermore, it produced a suitable vehicle with optimal interfacial surface tension and nano-size particles [15], in addition to the absence of precipitation [8]. Subsequently, characterization and further *in vitro* and *in vivo* investigation of the bioactivity study were undertaken for the delivery systems prepared using the 13 kDa LMWC.

### 2.4. Characterization of I-LMWC PEC and I-LMWC RM Systems

The particle size results for I-LMWC PECs prepared using the same insulin concentration (3.5 mg/mL) and different concentrations of 13 kDa LMWC (1–5 mg/mL) were compared with the particle size results for insulin and LMWC alone. The particle size of insulin in solution was found to be around 5.0 nm, which indicates that it is present in its hexameric form [35]. However, the particle size of LMWC is concentration dependent. When the LMWC concentration is low, LMWC particles are present in a more extended form; however, above a certain concentration aggregates start to form and the particle size starts to increase; similar results have been previously reported [36].

**Figure 3:**
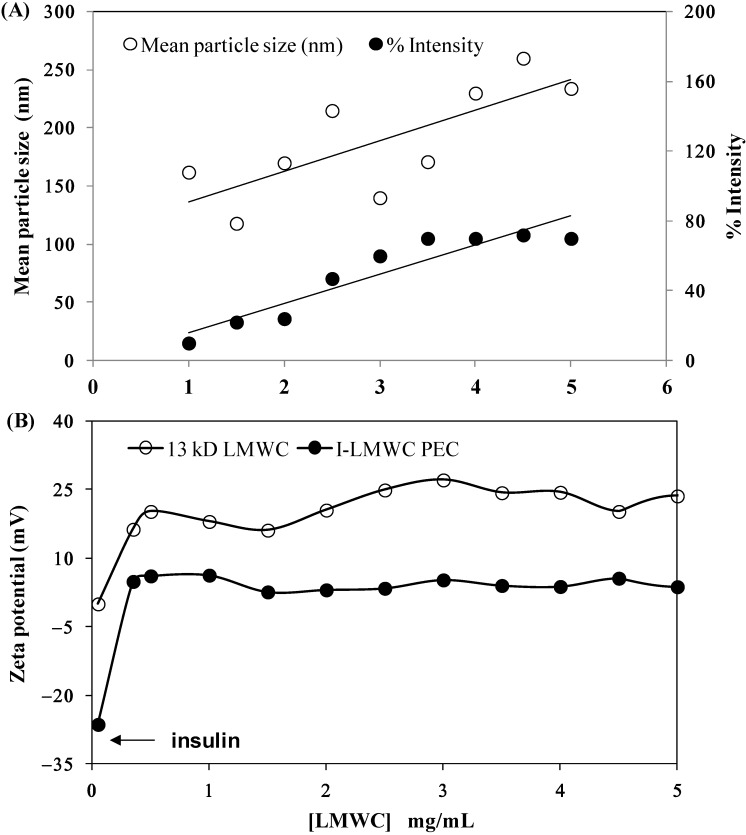
Effect of 13 kDa LMWC concentration on (**A**) the particle size of major peaks at 100–250 nm and (**B**) the zeta potential of the insulin–LMWC polyelectrolyte (I-LMWC PEC). The number of replicates is 6 and 3, respectively.

At low concentration of LMWC (1 mg/mL) the size distribution intensity of I-LMWC PECs was 6.38 ± 0.26 nm (higher than the size of insulin), and only one peak was present when measuring particle size. This means that all the LMWC molecules interact with insulin molecules to form a particle larger than insulin. Increasing the LMWC concentration to 3.5 and 5 mg/mL resulted in a particle size of 6.9 ± 0.17 nm; however, another peak started to appear in the region of around 100 ± 20 nm, which represents the size of the free LMWC and aggregated LMWC in the sample (*i.e.*, LMWC un-reacted with insulin). The results of mean particle size are shown in Figure 3A. Since LMWC will react with insulin at one site only, an excess amount of LMWC will be free in the sample that may also aggregate and give a particle size of around 100 nm. The mean particle size of the RMs was 300 ± 19 nm.

The effect of LMWC concentration on the zeta potential of I-LMWC PEC (3.5 mg insulin/mL) was investigated. LMWC (1–5 mg/mL) without associated insulin shows a positive zeta-potential of +15–+25 mV (Figure 3A). On the other hand, the presence of insulin (with a zeta-potential of −27 mV) decreases the zeta-potential value to about +5 mV. The ionized groups of amino acid residues of insulin will be attracted to the positively charged LMWC via coulombic forces [37]. The zeta potential of the complex is positive, which means that insulin is encapsulated in the polymer, projecting positively charged chains towards the external aqueous medium. Although the zeta potential value of the complex is positive, the surface charge of the complex will be decreased when compared to LMWC alone, which is expected. This facilitates absorption of the PEC to the biolipid membranes of the GIT [38].

The data for the effect of 13 kDa LMWC concentration and pH of the I-LMWC PEC on insulin association efficiency (AE) are presented in Table 4. It is well known that the formation of PEC is pH dependent [8,39]. The investigated pH range (6.5–7.0) in the present work is close to the pK_a_ of chitosan (about 6.5) and to the isoelectric point (pI) of insulin (about 6.4). In this pH range, electrostatic, hydrophobic interactions as well as hydrogen bonding may be involved in PEC formation, as neutral and ionized chitosan species exist in an almost equal proportion [40].

**Table 4:** Effect of 13 kDa LMWC concentration and pH on insulin association efficiency (AE).

LMWC Concentration (mg/mL)	pH_f_	AE ± RSD (%)
0.7	6.5	63.8 ± 2.1
3.0	6.5	76.2 ± 3.2
0.7	6.7	22.7 ± 2.8
3.0	7.0	8.6 ± 4.6

Number of replicates is 3 for each experiment.

In the present work, the PEC using 13 kDa LMWC was prepared at different pH values of 6.5, 6.7, and 7.0. However, lower and high pH values were not considered (8 < pH < 5), where insulin degrades rapidly [8,41]. A change in pH from 6.5 to 7.0 results in a significant decrease in the value of the AE (from 76.5% to 8.6%) (Table 4). This may indicate that the PEC begins to precipitate at pH values higher than 6.5 as a result of a decrease in the solubility of the 13 kDa LMWC above its pk_a_ value (about 6.5) [8,42]. Such precipitation was observed for aqueous solutions of 13 kDa LMWC following pH adjustment to 7.0. This noticeable precipitation is probably due to the formation of neutral LMWC. However, the AE was improved by using higher concentrations of LMWC. Such behavior was previously observed by Wu *et al.* [43].

### 2.5. In Vitro Evaluation of I-LMWC PEC and I-LMWC RM

#### 2.5.1. *In Vitro* Release

**Figure 4:**
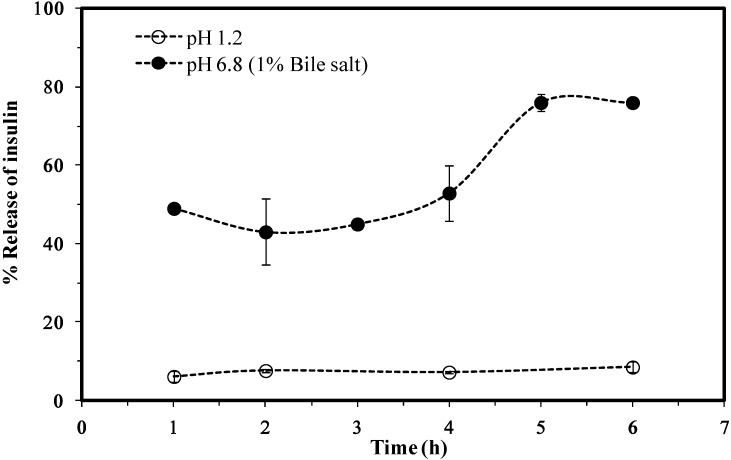
A cumulative release of insulin from insulin–LMWC reverse micelles (I-LMWC RMs) using 13 kDa LMWC.

The results for the *in vitro* release of insulin from the I-LMWC RM system showed a negligible release at pH 1.2 (<10% at 6 h), while at pH 6.8 the release is markedly increased (80% of the encapsulated amount at 6 h). Furthermore, the release profile at pH 6.8 was gradual and free of any detectable burst effects, as shown in the data in Figure 4.

#### 2.5.2. *In Vitro* Evaluation of the Protective Effect

The protective ability of the RMs under conditions simulating the gastric environment was evaluated and compared with free insulin and I-LMWC PEC. Free insulin and I-LMWC PEC were found to be completely degraded during incubation with pepsin, while in the RM about 90% of insulin was recovered after incubation with simulated gastric fluid with pepsin. These results emphasize the importance of the RM system in the protection of insulin from degradation by pepsin.

### 2.6. Biological Activity

**Figure 5:**
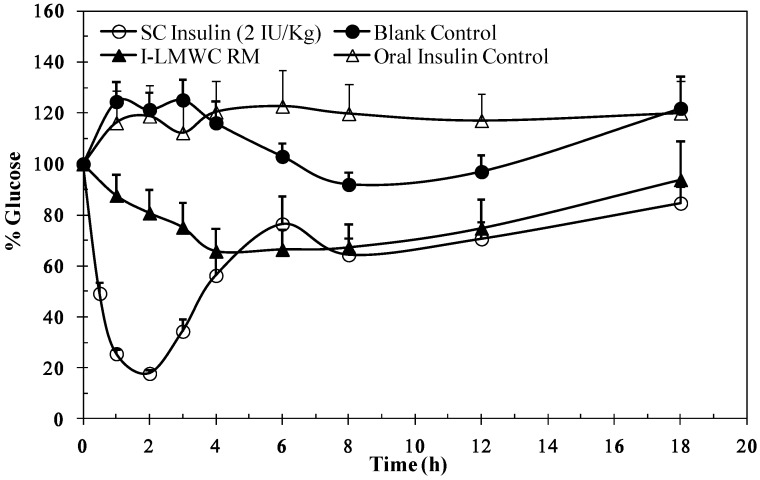
Blood glucose levels *versus* time profile after a single oral administration of 50 IU/kg insulin-LMWC reverse micelle (I-LMWC RM) and a subcutaneous administration (SC) of 2 IU/kg insulin to STZ-diabetic rats The results are expressed as mean ± SD (*n* = 10 for each group).

The data in Figure 5 illustrate the changes in blood glucose levels of rats after oral administration of I-LMWC PEC after solubilization in the reverse micelles. A decrease in plasma glucose level was observed. The results are significantly different when compared to a blank control (*p* < 0.05). The reverse micelle preparation resulted in minimum glucose levels, about 70% after 3 h, and the reduction in glucose levels was maintained over a prolonged period of time. It is worth mentioning that insulin and I-LMWC PEC are completely degraded under conditions simulating the gastric environment, as reported previously [14]. However, the decrease in blood glucose levels (Figure 5) may be attributed to the improved stability of insulin in the reverse micelle preparation against degradation at gastric pH values and in the presence of enzymes in the GIT system.

## 3. Experimental Section

### 3.1. Materials

USP human insulin RS (26.4 USP insulin human units/mg, lot No. J0J250) was purchased from USP Convention (Rockville, MD, USA). Recombinant human (rh) insulin of pharmaceutical grade standardized by using USP insulin human RS (potency 99.4%) was purchased from Biocon (Bangalore, India). Purified water and LMWCs of different molecular weights (1.3, 6, 13, 18, and 30 kDa) with >95% degree of deacetylation (DDA) were provided by the Jordanian Pharmaceutical Manufacturing Company (Naor, Jordan). LMWCs may be considered as derivative of chitosan with lower molecular weight, obtained by depolymerization of high molecular weight chitosan HCl (≈250 kDa and DDA 95%, Xiamen Xing, Shanghi, China). Oleic acid of pharmaceutical grade (purity >99%) was purchased from Merck (Darmstadt, Germany). Labrasol^®^ (PEG-8 caprylic/capric glycerides) and Plurol^®^Oleique CG (polyglycerol-6-dioleate) were purchased from Gattefosse (Saint-Priest, Lyon, France). Streptozotocin was purchased from Sigma-Aldrich (St. Louis, MO, USA). All other chemicals were of analytical/HPLC grade and purchased from Merck.

### 3.2. Determination of Molecular Weight of LMWCs

The average molecular weight of LMWCs was determined by viscosity measurements (Vibro viscometer, SV-10 Japan) in water. The viscosity average molecular weight was obtained using the Mark–Houwink’s equation, [η] = k·M^a^, where [η] is the intrinsic viscosity, M is the viscosity average molecular weight, and the k and a values were 0.00058 and 0.69, respectively, based on a previous study [44].

### 3.3. Preparation of I-LMWC PEC

LMWC (1.3, 6, 13, 18, and 30 kDa) solutions of the required concentration (0.3–7 mg/mL) were prepared by dissolving LMWC powder in water, adjusting the pH to 5.5 with 0.1 N NaOH, and then making up to the required volume with water.

Insulin solutions (7 mg insulin/mL) were prepared by dissolving insulin powder in 1 mL of 0.1 N HCl, neutralized with 0.1 N NaOH and made up to a volume of 10 mL with water or 1 M Tris (hydroxylmethyl-aminomethane) buffer, pH 6.5.

I-LMWC PEC was prepared by mixing equal volumes of LMWC and insulin solutions in a glass vial under gentle magnetic stirring and incubating for 10 min at room temperature. I-LMWC PEC preparations contained 3.5 mg insulin/mL and different concentrations of LMWC (0.0–3.5 mg/mL).

### 3.4. Preparation of I-LMWC RM

An RM system was prepared based on a previous study [13]. A surfactant mixture of Labrasol^®^ and Plurol^®^ Oleique CG was prepared at a 1/1 (w/w) ratio by mixing the constituents using magnetic stirring for 5 min. Oleic acid (80 g) and surfactant mixture (20 g) were mixed together for 5 min; 8 mL of I-LMWC PEC (3.5/1.5), which contained 3.5 mg insulin and 1.5 mg 13 kDa LMWC/mL, was added to the mixture and mixed using a magnetic stirrer (250 rpm for 5 min).

### 3.5. Extraction Procedures

For the I-LMWC PEC system, equal volumes of the sample and a solvent mixture of 0.01 N HCl and methanol (2/3, v/v) were mixed together.

For the reverse micelle system, 2 g of the sample and 4 mL of the solvent mixture were vortexed vigorously for 2 min, centrifuged at 4000 rpm for 15 min, and the aqueous phase collected. The samples were then analyzed by HPLC to determine the insulin, A-21 desamido insulin, and HMWP content (3 replicates); the RSD values were <5%, <0.5%, and <3%, respectively.

### 3.6. HPLC Determination of Insulin

Quantitative determination of insulin was based on the USP assay method [16]. The HPLC system consisted of a TSP 1000 pump system, a TSP 1000 UV-VIS detector, and a TSP AS 3000 auto-sampler (TSP, USA). A C18 (L1) column (particle size 5 µm), dimensions of 4.6 × 150 mm (Thermo column from Thermo Fisher Scientific Inc., Rockford, IL, USA), maintained at 40 °C during analysis, was used as the stationary phase, together with a Lichrospher 100 RP-18, 5 µm particle size guard column (Merck, Germany). Elution was performed isocratically (flow rate 1 mL/min) using sulfate buffer pH 2.3-acetonitrile (73/27, v/v) as the mobile phase and UV detection at 214 nm. The injection volume was 20 μL. The resolution, R, between insulin and A-21 desamido insulin was >2, the tailing factor for insulin peak was <1.8, and RSD was <1.6%.

The resolution solution was prepared by dissolving 1.5 mg of insulin in 1 mL of 0.01 N HCl, followed by incubation of the solution at room temperature for not less than 3 days. Equipment control, data acquisition, and integration were undertaken using a ChromQuest work station.

### 3.7. Size Exclusion HPLC Determination of High Molecular Weight Proteins (HMWPs)

The method used was based on the USP limit test for HMWPs [16]. A column containing dihdroxypropane bound to silica packing material (L20) with a particle size of 5 μm and dimensions of 7.8 × 300 mm was used as the stationary phase (Waters insulin HMWP column, Dublin, Ireland). Elution was performed isocratically (flow rate 0.5 mL/min) using a mixture of an arginine solution (1 mg/mL), acetonitrile, and glacial acetic acid (65/20/15, v/v) as the mobile phase and UV detection at 276 nm. The injection volume was 100 μL.

Resolution solution was prepared by dissolving 4 mg of insulin containing not less than 0.4% HMWPs in 1 mL of 0.01 N HCl (insulin containing the indicated fraction of HMWPs was prepared by allowing insulin powder to stand at room temperature for about 5 days) [16].

### 3.8. HPLC Method Verification

The two USP HPLC methods (insulin assay and limit test for HMWPs) were verified according to USP [45] and the International Conference of Harmonization (ICH) guidelines [46]. Standard insulin solutions of different concentrations (0.9, 1.75, 3.5, 7.0, and 10.0 mg/mL) were used to assess the linearity of the calibration plot (3 replicates). The precision of the assay was determined by analyzing samples of I-LMWC PEC preparations at three different insulin concentrations (1.0, 3.5, and 10.0 mg/mL). For the assessment of the inter-day variation, samples were analyzed in triplicate (*n* = 3) on three different days. For the intra-day variation, they were analyzed 5 times (*n* = 5) on the same day. Accuracy was assessed by analyzing samples of I-CCS PEC preparations at the target concentrations of insulin of 3.5 mg/mL. Specificity was verified by analyzing the matrix, *i.e.*, LMWC PEC, in the absence of insulin using the extraction method stated above. Additionally, a resolution solution containing insulin and A-21 desamido insulin was prepared and injected. The detection limit (DL) and quantitation limit (QL) for the HPLC method were determined based on the standard deviation of the response and the slope of the calibration curve, respectively. The stability of solutions of insulin (0.9 mg/mL) was analyzed by HPLC at 0, 12, and 24 h of storage at room temperature.

### 3.9. Stability of I-LMWC PEC

The initial experiments were designed to incubate the I-LMWC PEC samples at 40 °C; however, as long periods of time (>2 months) were required to obtain indicative results, the experimental design was changed and the samples were incubated at a higher temperature (50 °C). Samples of I-LMWC PEC preparations, using LMWCs of different molecular weights and concentrations, were incubated at 50 °C for different periods of time. Samples were withdrawn at pre-determined intervals (0, 12, 24, 36, 48, and 72 h) and tested for insulin, A-21 desamido insulin, and HMWP content and the physical stability of I-LMWC PEC preparations. In addition, the stability of insulin at different concentrations (0.9–10 mg/mL) in water and Tris-buffer at pH 6.5 was investigated at 50 °C.

### 3.10. Immunological Bioactivity of Insulin

Enzyme-linked immunosorbent assay (ELISA) was used to assess the immunological stability of insulin following formulation. Active insulin ELISA DSL-10-1600 micro-titration kits (Diagnostic Systems Laboratories Inc., Webster, TX, USA) were used. Insulin concentration was measured using an enzymatically amplified “one-step” sandwich–type immunoassay. The samples were incubated with an anti-insulin antibody in micro-titration wells that had been coated with another anti-insulin antibody. Insulin was extracted from the I-LMWC PEC (3.5/1.5 mg/mL) preparations of different molecular weight (1.3, 6, 13, 18, and 30 kDa), as described above, and then assayed according to the instructions of the manufacturer. The results were obtained by reading the optical density at 450 nm with background wavelength correction at 620 nm using a Bio-Rad microplate reader (Bio-Rad, Hercules, CA, USA).

### 3.11. Characterization of I-LMWC PEC

Particle size measurements were undertaken by dynamic light scattering using a Zetasizer Nano ZS instrument (Malvern, UK). Replicate measurements (*n* = 6) were carried out at 25 °C using a detection angle of 90°.

Zeta potential measurements (*n* = 3) were also carried out using the Zetasizer Nano ZS instrument at 25 °C using folded capillary cells integrated with gold electrodes.

I-LMWC PECs were centrifuged at 14,000 rpm for 30 min at room temperature. The quantity of insulin in the supernatants was measured using HPLC and the insulin association efficiency was calculated accordingly [14]. The number of replicates was 3 for each experiment.

### 3.12. In Vitro Evaluation of I-LMWC PEC and I-LMWC RM

#### 3.12.1. *In Vitro* Release

The *in vitro* release studies of insulin from I-LMWC RM were performed by incubating in a simulated gastric medium at pH 1.2 and a simulated intestinal medium, pH 6.8, (1% bile salt v/w) at 37 °C under continuous shaking at 50 rpm. Samples were withdrawn at specific time intervals (1, 2, 3, 4, 5, and 6 h), centrifuged, and analyzed for insulin release using the USP HPLC method [16].

#### 3.12.2. *In Vitro* Evaluation of the Protective Effect

A sample of 5 mL simulated gastric fluid (SGF) with pepsin (pH 1.2) was added to a 1 mL sample of free insulin solution, I-LMWC PEC solution, and a 2.0 g sample of I-LMWC PEC solubilized in the RM system. The samples were incubated for 1 h at 37 °C while shaking at 100 strokes/min. A 1.5 g sample of the RM was mixed with 5 mL of extraction solution of 0.010 M HCl and methanol (2/3, v/v), vortexed for 3 min, and a 100 µL sample of the aqueous layer was analyzed for insulin content by HPLC [16]. For insulin and I-LMWC PEC solutions, 100 µL samples were withdrawn after 1 h and analyzed for insulin content.

### 3.13. In Vivo Pharmacological Activity

Animal studies were conducted using adult male Sprague Dawley rats (body weight range 250–300 g) randomized into groups (*n* = 10). The animals were housed in air conditioned quarters under a photoperiod schedule of 12 h light/12 h dark cycles. The rats received standard laboratory chow and tap water 3 weeks prior to the experiments. Animal care and use was performed in compliance with the guidelines of the Federation of European Laboratory Animal Science Association and European Union (Council Directive 86/609/EEC). The study protocol was approved by the Ethical Committee of the Jordanian Pharmaceutical Manufacturing Company (JPM), Naor, Jordan. Diabetes was induced in male Sprague Dawley rats by intraperitoneal injection of two doses of 80 mg/kg streptozotocin (STZ) over two days. STZ was freshly prepared by dissolving in 0.1 M citrate buffer, pH 4.5. Blood glucose levels were monitored by measuring the glucose concentrations in blood samples obtained from the tail vein of rats using a blood glucose meter (Gluco Dr. All Medicus, Korea, range 112–444 mg/dL). Only rats with a basal blood glucose level above 200 mg/dL were considered diabetic.

STZ diabetic rats (*n* = 10) were randomized into different groups. Following initial blood glucose determinations, one group was injected with 2 IU/kg insulin and served as a positive control for insulin bioactivity. The other groups were given single an oral dose administration (50 IU/kg) of a blank control (same RM components, but without insulin), oral insulin solution, and I-LMWC RM. Blood sampling for glucose measurements proceeded during the experiments at specific time intervals (1, 2, 3, 4, 5, 6, 8, 12, and 18 h) post insulin administration.

## 4. Conclusions

The insulin USP compendium method for insulin analysis and HMWPs can be used to evaluate insulin stability in a delivery system containing insulin as well as I-LMWC PEC. Insulin stability was evaluated for insulin content, formation of HMWPs, and biological activity. In the literature, minimal studies are available covering these three elements, especially formation of HMWPs, which are very important in evaluating the suitability of the delivery system. Extraction procedures must be carefully carried out in order not to affect insulin results. Retention time optimization is important and currently insulin analysis can be executed within 20–30 min with such a convenient HPLC method. Based on stability studies, solutions of 3.5 mg/mL insulin and 1.5 mg/mL 13 kDa LMWC constitute the optimum stability formulation of the I-LMWC PEC for orally delivered insulin. Our experimental design allowed for the optimization of insulin formulation by determining parameters that affect the chemical and physical stability of insulin. The I-LMWC PEC preparation method has the advantage of not necessitating sonication and use of organic solvents during preparation, thereby minimizing possible degradation of insulin. The I-LMWC PEC system has been characterized in terms of insulin stability, where the study revealed that complexation with LMWC improves the stability of insulin. Both ELISA and *in vivo* studies confirmed the bioactivity of loaded insulin in the delivery system examined.
